# Decreased Heart Rate Variability in HIV Positive Patients Receiving Antiretroviral Therapy: Importance of Blood Glucose and Cholesterol

**DOI:** 10.1371/journal.pone.0020196

**Published:** 2011-05-31

**Authors:** Gro Askgaard, Ulrik Sloth Kristoffersen, Jesper Mehlsen, Gitte Kronborg, Andreas Kjaer, Anne-Mette Lebech

**Affiliations:** 1 Department of Infectious Diseases, Hvidovre University Hospital, Hvidovre, Denmark; 2 Clinic of Clinical Physiology, Nuclear Medicine & PET, Rigshospitalet, Copenhagen, Denmark; 3 Cluster for Molecular Imaging, University of Copenhagen, Copenhagen, Denmark; 4 Department of Clinical Physiology & Nuclear Medicine, Frederiksberg University Hospital, Frederiksberg, Denmark; Mayo Clinic, United States of America

## Abstract

The presence of autonomic dysfunction in HIV patients is largely unknown. Early studies found autonomic dysfunction in patients with AIDS. Antiretroviral combination therapy (ART) has dramatically changed the course of the disease and improved prognosis and decreased morbidity.

**Aim:**

To evaluate whether autonomic dysfunction is present in an ART treated HIV population and if so to identify factors of importance.

**Methods:**

HIV patients receiving ART for at least 12 months (n = 97) and an age-matched control group of healthy volunteers (n = 52) were included. All were non-diabetic and had never received medication for hypertension. Following a 10 min resting period a 15 min ECG recording was performed. Heart-rate variability (HRV) analysis was performed in accordance with current guidelines and data reported as mean [interquartile range].

**Results:**

Mean normal-to-normal (NN) and total HRV measured as standard deviation of normal-to-normal (SDNN) was lower in HIV patients compared to controls (905 vs. 982 ms; p<0.001 and 48 vs. 54 ms; p = 0.028, respectively). No differences were found between the groups in parasympathetic activity measured as square root of the mean squared difference of successive NN-intervals (RMSSD) or the percent of differences between adjacent NN intervals greater than 50 ms (pNN50). In the HIV positives, haemoglobin A1c correlated inversely with SDNN, RMSSD and pNN50 (p<0.05). Total cholesterol and LDL-C correlated inversely with RMSSD and pNN50 (p<0.05). Neither HIV duration, HIV-RNA, CD4 cell count nor CD4 nadir correlated with time or phase domain HRV variables.

**Conclusions:**

Moderate autonomic dysfunction is present in HIV positives patients even with suppressed viral load due to ART. The dysfunction is correlated with HbA1c and hypercholesterolemia but not to duration of HIV or whether the patients were receiving protease inhibitors as part of the ART regime.

## Introduction

Various markers have been used to identify autonomic dysfunction (AD) including decreased heart rate variability (HRV) and delayed heart rate recovery (HRR) after exercise. Impaired autonomic function has been found to predict increased risk for cardiac events and sudden death in patients with cardiac disease as well as in apparently healthy people [1]–[4].

Autonomic dysfunction has been reported in patients with HIV [5]–[11]. The prevalence seems to be particularly high in patients with advanced disease [12], [13]. Most studies have been performed in untreated patients or patients with very advanced disease. However, the availability of potent combination antiretroviral regimes (ART) has resulted in a dramatic reduction in HIV-associated morbidity and mortality [14]. It would therefore be of interest to study the prevalence of autonomic dysfunction in an ART treated HIV population. We speculate that suppression of HIV virus due to ART could reduce the prevalence of autonomic dysfunction. However, ART is also known to induce an array of adverse effects, among them dyslipidemia and insulin resistance [15]–[17]; and specifically ART regimens including a protease inhibitor (PI) has been linked to development of hyperglycemia and hyperlipidemia in some HIV infected patients.

Since diabetes is a known cause of autonomic dysfunction the diabetogenic effect of ART could cause autonomic dysfunction in HIV patients in ART. Furthermore several antiviral drugs have been associated with development of toxic neuropathy including nucleoside analogue reverse transcriptase inhibitors as didanosine and stavudine [18], [19].

Therefore, ART associated autonomic dysfunction could in principal be caused both by the diabetogenic and the neurotoxic effect among other unrecognized causes.

Recently, in a small pilot study we found indications of existence of autonomic dysfunction despite optimal treatment of HIV patients [6]. However, a larger study is needed to confirm these findings as well as to gain the statistical power that allows for analysis of possible causative relations, e.g. changes in lipids and blood glucose as well as influence of different components of ART. The present study was designed to allow for analysis of such correlations.

The aim of the present study was therefore to study the presence of autonomic dysfunction in a population of HIV positive patients receiving ART. In addition, factors influencing HRV in HIV positive patients were also studied.

## Materials and Methods

### Subjects

Between 2004 and 2009 patients and controls were enrolled. The Danish Scientific Ethical Committee approved the study (No. H-C-2009-051) and written informed consent was obtained from all the participants.

#### HIV patients

A total of 97 patients with HIV infection were included. The patients were recruited from the outpatient clinic at the Department of Infectious Diseases, Hvidovre University Hospital, Copenhagen, Denmark. Eighty-six (83%) were Caucasians and 11 (11%) were Africans, 6 (6%) were Asian and 1 (1%) Inuit. Inclusion criteria were: (i) age 18 years or older, (ii) proven HIV infection, (iii) receiving stable doses of ART for ≥12 months. Exclusion criteria: (i) clinical history of cardiovascular or ischemic heart disease, (ii) known alcohol abuse, (iii) diabetes mellitus, (iv) medication for hypertension or (v) pregnancy.

The patients had been HIV positive for median 11.1 years. Twenty (19%) of the HIV patients had been diagnosed with AIDS. Patient characteristics including biochemical data are shown in table 1.

**Table 1:** Comparison of study groups.

	Control group	HIV group	Significance
Number of subjects	52	97	
Gender (male/female)	43/9	75/29	NS^a^
Age (years)	49 (43–56)	46 (40–54)	NS^b^
Body mass index (kg/m^2^)	25 (24–27)	25 (22–27)	NS^b^
Systolic blood pressure (mmHg)	123 (116–135)	125 (115–140)	NS^b^
Diastolic blood pressure (mmHg)	74 (68–81)	79 (71–89)	p = 0.03^b^
Hemoglobin (mmol/L)	9.1 (8.7–9.2)	8.7 (8.3–9.3)	p = 0.03^b^
Creatinine (mmol/L)	77 (68–81)	73 (66–83)	NS^b^
Glucose, blood (mmol/L)	5.2 (4.8–5.5)	5.5 (5.1–5.9)	p = 0.004^b^
Hemoglobin A1c (%; DCCT)	5.6 (5.4–5.8)	5.2 (5.0–5.6)	p<0.001^b^
Cholesterol, total (mmol/L)	5.1 (4.5–5.6)	5.5 (4.6–6.3)	NS^b^
HDL-cholesterol (mmol/L)	5.1 (4.5–5.6)	1.3 (1.0–1.5)	NS^b^
LDL-cholesterol (mmol/L)	5.1 (4.5–5.6)	3.1 (2.4–4.0)	NS^b^
Triglyceride (mmol/L)	5.1 (4.5–5.6)	1.6 (1.0–2.2)	p<0.001^b^

Values are median *(interquartile range).*

a) Fishers exact test;

b) Mann-Whitney test.

#### Controls

Fifty-two age and gender matched healthy volunteers were included. Their basic characteristics are summarized in table 1. The control subjects were included from individuals participating in a Danish prospective population study (Copenhagen Centre of Prospective Population Studies at the Institute of Preventive Medicine, Bispebjerg Hospital, University of Copenhagen, Denmark). All were Caucasians.

### Heart rate variability testing

Following a 10 min resting period a 15 min ECG recording was performed while patients were in the supine position. HRV analysis was performed in accordance with current international guidelines [20]. The ECG data were sampled by a Holter monitoring recorder at 250 kHz and analysed by the HolterSoft Ultima soft-ware (Novacor SA, Cedex, France) that complies with current guidelines. The time domain analysis variables were: mean normal-to-normal (NN; units: ms), standard deviation of NN (SDNN; units: ms) and square root of the mean squared difference of successive NN-intervals (RMSSD; units: ms) and the percent of differences between adjacent NN intervals greater than 50 ms (pNN50). SDNN is considered an estimate of overall HRV and thus both an indicator of sympathetic and parasympathetic influence. RMSSD is an estimate of short-term components of HRV and thus considered reflecting mainly the parasympathetic influence. Deterministic analysis was performed as Poincaré plot analysis, also known as Lorenz plot analysis. The variables were: L_L_ (length of Lorenz plot), L_B_ (width of Lorenz plot) and L_SD_ (difference between geometric and mass center). L_L_ is considered an indicator of long-term variability and L_B_ the short-term variability.

### Statistical analysis

We performed Kolmogorov-Smirnov test of all variables to test for assumption of normal distribution. Most of the HRV measures as well as most of the biochemical markers were not normal distributed. Accordingly, the data were handled using non-parametric statistics. Therefore, data are presented as median with 25 and 75 percentiles in parenthesis. Comparisons between the groups were performed using Fisher's exact test for binomial data (2×2 contingency tables) and Mann-Whitney test for continuous data. Correlations were analyzed using non-parametric (Spearman) correlation and expressed with Spearman's rho. P<0.05 was considered significant. P-values between 0.05 and 0.1 was considered borderline significant.

## Results

The group of HIV positives did not differ from the control group with respect to age, gender, body mass index, systolic blood pressure, creatinine and cholesterol (including HDL-C and LDL-C). The HIV patients as a group had lower haemoglobin, higher blood glucose and higher triglyceride. The data are summarized in table 1.

The HIV parameters and the ART of the HIV study group are summarized in table 2. Median duration of HIV was 11.1 years and median duration of ART was 7 years.

**Table 2:** HIV parameters of study group (n = 97).

Duration of HIV (years)	11.1 (5.8–15.6)
Duration of ART (years)	7.2 (3.6–11.8)
CD4 (cells/mm^3^)	552 (434–717)
CD4 nadir (cells/mm^3^)	200 (98–260)
ART	
3 NRTI	5 (5%)
2 NRTI + 1 NNRTI	59 (61%)
2 NRTI + 1 PI	26 (27%)
1 NNRTI + 1 PI	7 (7%)
Fully suppressed HIV RNA*	96/97 (99%)**

Values are median *(interquartile range)*.

ART: Highly active antiretroviral therapy; NRTI: Nucleoside transcriptase inhibitor, NNRTI: Non-nucleoside transcriptase inhibitor, PI: Protease inhibitor.

*) HIV RNA≤400 copies/mL;

**) The remaining patient had viral load = 560 copies/ml.

### Heart rate variability: Time domain analysis

Mean NN was significantly lower in HIV patients compared to the controls (905 vs. 982 ms; p<0.001). SDNN was significantly lower in the HIV group compared to the controls (48 vs. 54 ms; p = 0.042). No differences were found between the groups regarding RMSSD and pNN50. Data of the time domain analysis are summarized in table 3.

**Table 3:** Heart-rate variability: Time domain analysis.

	Control group (n = 52)	HIV group (n = 52)	HIV group
Mean NN (ms)	982 (894–1,103)	905 (850–989)	p<0.001
SDNN (ms)	54 (43–72)	48 (37–65)	p = 0.042
RMSSD (ms)	28 (22–41)	29 (20–49)	NS
pNN50 (%)	5 (1–9)	5 (1–20)	NS

Values are median *(25 percentile-75 percentile).*

Mean NN: mean normal-to-normal, SDNN: Standard deviation of NN; RMSSD: Square root of the mean squared difference of successive NN-intervals; pNN50: Percent of differences between adjacent NN intervals greater than 50 ms.

Statistical comparison was performed by Mann-Whitney test.

#### Correlation with biochemical parameters

**Figure 1:**
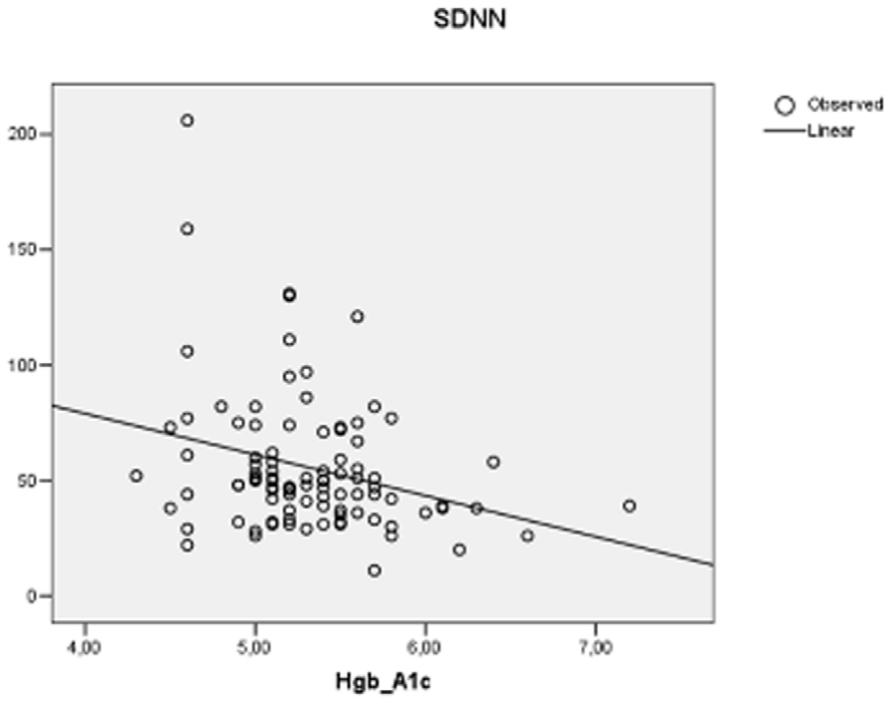
Correlation between Hgb A1c and standard deviation of NN (SDNN; units: ms) in 97 HIV positive patients receiving antiretroviral treatment.

**Figure 2:**
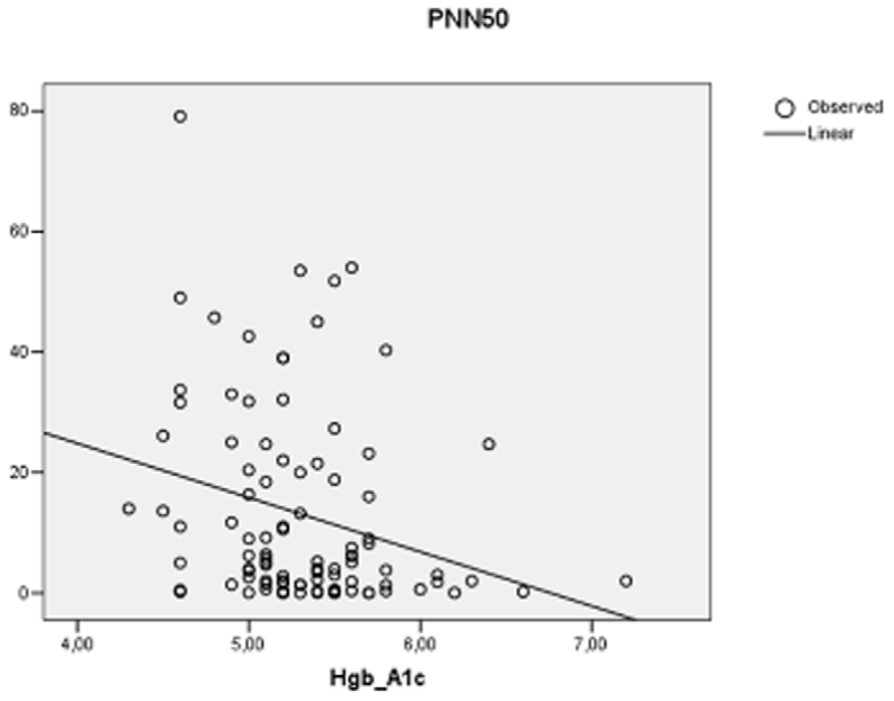
Correlation between Hgb A1c and percent of differences between adjacent NN intervals greater than 50 ms (PNN50) in 97 HIV positive patients receiving antiretroviral treatment.

In the HIV positive group haemoglobin A1c correlated inversely with SDNN (r = −0.234; p = 0.027; Fig. 1), RMSSD (r = −0.287; p = 0.006) and pNN50 (r = −0.299; p = 0.003; Fig. 2). In addition, in HIV positives total cholesterol inversely correlated with RMSSD (r = −0.259; p = 0.011) and pNN50 (r = −0.237; p = 0.021) but not with mean NN or SDNN. Likewise, LDL-cholesterol inversely correlated with RMSSD (r = −0.262; p = 0.012) and pNN50 (r = −0.250; p = 0.017) but not with mean NN or SDNN. In the control group no correlations were found between the biochemical parameters and time domain variables.

#### Correlation with HIV parameters

None of the HIV parameters, HIV duration, HIV-RNA viral load, CD4 cell count or CD4 nadir, correlated with any of the time domain HRV variables.

#### Effect of HIV medication

Duration of ART was between 3.6 years and 11.8 years. There was a borderline significant inverse correlation between ART duration and mean NN (r = −0.171; p = 0.093). Of the 97 HIV patients, 33 patients (34%) received protease inhibitors (PI) whereas 64 (66%) did not. No difference was found between the group receiving PI and the one not receiving a PI-sparing regime and mean NN, SDNN, RMSSD or pNN50. Likewise no difference was found when patients in ART were stratified on basis of receiving abacavir (n = 55; 57%) or not receiving abacavir (n = 42; 43%) in mean NN, SDNN, RMSSD or pNN50. Only 7 patients received didanosine and none of the patients stavudine. Thus statistical analyses were not performed regarding these antiviral drugs typically associated with development of toxic neuropathy.

### Heart rate variability: Poincaré plot analysis (phase domain analysis)

No differences were found between HIV patients and control in any of the 3 variables L_L_, L_B_ and L_SD_. Data on Poincaré plot analysis are summarized in table 4.

**Table 4:** Heart-rate variability: Poincaré plot analysis (phase domain).

	Control group (n = 52)	HIV group (n = 97)	Significance
L_L_ (ms)	360 (188–481)	353 (255–445)	NS
L_B_ (ms)	131 (110–148)	130 (121–153)	NS
L_SD_ (ms)	20 (8–34)	19 (10–27)	NS

Values are median *(25 percentile-75 percentile)*.

L_L:_ Length of Lorenz plot; L_B_: Width of Lorenz plot; and L_SD_: Difference between geometric and mass center.

Statistical comparison was performed by Mann-Whitney test.

#### Correlation with biochemical parameters

In the HIV positive group haemoglobin A1c correlated inversely with L_B_ (r = −0.209; p = 0.049) but not with L_L_ or L_SD_. No other biochemical parameters measured (see table 1) correlated with L_B_, L_L_ or L_SD_. In the control group no correlations were found between the biochemical parameters and phase domain variables.

#### Correlation with HIV parameters

None of the HIV parameters, HIV duration, HIV-RNA, CD4 or CD4 nadir, correlated with L_B_, L_L_ or L_SD_.

#### Effect of HIV medication

There was a borderline significant inverse correlation between ART duration and L_B_ (r = −0.188; p = 0.066) but not with L_L_ or L_SD_. No difference was found in any of the phase domain variables between the group receiving or not receiving PI. Likewise no difference was found between patients receiving or not receiving abacavir.

## Discussion

The main finding in our study was presence of autonomic dysfunction in a group of HIV patients with suppressed viral load due to ART when compared to age and gender matched healthy HIV negative controls.

We found a decrease in mean NN, i.e. an increased resting heart rate probably indicating a decrease mainly in parasympathetic tone since it has a strong influence on the resting heart rate. In addition, a lower SDNN was found in the HIV patients also indicating an overall decrease in heart rate variability since SDNN is considered an indicator of overall HRV, i.e. the combined parasympathetic and sympathetic input. Taken together it therefore seems likely that at least the parasympathetic input is blunted. However, the indicators of short-term variability that normally are considered to primarily reflect the parasympathetic regulation, RMSSD and L_B_ were not different between the groups indicating that the dysfunction may not only be parasympathetic.

The present results are in accordance with our previous study of 16 ART treated HIV patients where reduced HRV was found as well [6]. In studies performed prior to effective treatment regimes patients with advanced HIV disease showed in general both sympathetic and parasympathetic dysfunction. The autonomic dysfunction in untreated patients with advanced disease was generally believed to be caused by HIV-1 virus itself, which is well known to be neurotropic [12], [21]. Moreover immune suppression may also be responsible for development of autonomic dysfunction. However, in our study we did not find any correlation between measures of HRV and HIV duration, HIV-RNA, CD4 cell count or CD4 nadir. In agreement with this, no statistical correlation was found between autonomic symptom scores and CD4 cell count in HIV positive treatment naïve African patients [22].

Since the introduction of ART it has been possible to treat HIV patients and when treated optimally to obtain continuous suppression of HIV RNA as well as high CD4-cell counts, which indicates immune competence. The treatment has decreased mortality of HIV patients but has also lead to long-term concern about possible adverse effects of treatment including greater risk for cardiovascular disease [23]. Adverse effect could be both due to the drugs themselves, and due to development of hypercholesterolemia, insulin resistance and metabolic syndrome, well known to be associated with ART [15]–[17]. However, in the present study we did not find any correlation between the HRV parameters and duration of ART. Since PI's have been linked to both hypercholesterolemia and development of insulin resistance, we also compared HIV patients receiving and not receiving PI as part of ART. Again, we did not find any difference in HRV measures. This finding is in agreement with a previous report that found impaired HRV in HIV positive individuals on ART whether or not the ART regime included a PI [10]. Recently, also abacavir in particular has been demonstrated to bear an increased risk of myocardial infarction [24]. Therefore, we also performed a comparison of patients receiving abacavir with those not receiving this drug. No difference in HRV was found.

Since development of autonomic dysfunction may be mediated via hypercholesterolemia and insulin resistance or hyperglycemia we compared the HRV parameters with biochemical measures. Here we found in the HIV group that a clear correlation existed between hemoglobin A1c and most of the HRV measures indicating that a high hemoglobin A1c predicted autonomic dysfunction. As hemoglobin A1c is a good parameter of long-term blood glucose it seems that high blood glucose is a risk factor in HIV patients for development of autonomic dysfunction and that this is a further reason to monitor blood glucose levels closely in this population. In contrast, no such correlation was found in the HIV negative group that in general had hemoglobin A1c levels of the same magnitude and range as the HIV patients. It therefore seems, that HIV patients somehow are more sensitive than HIV negatives to elevation in hemoglobin A1c. We have no obvious explanation for this but negative synergy with other factors in HIV-positive individuals may be a possible explanation. In future investigations in-depth study of on glucose metabolism, e.g. oral glucose tolerance test and measurement of insulin levels could be included to study the mechanism further. AD is a serious chronic complication of diabetes and in patients with diabetes-1 randomized prospective trials supports a central role for hyperglycemia in the pathogenesis of AD [25].

Also total cholesterol as well as LDL-cholesterol in the group of HIV positives correlated in our study with some of the HRV parameters. A high level of cholesterol or LDL-cholesterol was associated with decreased autonomic function measured as HRV. Again, this association was not found in the control group of HIV negatives even though the level and range of lipids were the same in the two groups. Therefore, also with regards to hypercholesterolemia it seems that it causes more damage to the autonomic nervous system in HIV patients than in HIV negatives. Again, the reason is not obvious but may be due to synergy with some other factors related to HIV disease. Also with regards to cholesterol levels it therefore seems more important to monitor and treat in HIV positives than in HIV negatives in order to prevent development of autonomic dysfunction.

In summary we found that autonomic dysfunction is present in HIV positives patients with suppressed viral load due to ART although the degree of dysfunction is moderate in most cases. The dysfunction is neither related to HIV duration, HIV-RNA, CD4 cell count, C4 nadir or ART duration nor to receiving PIs or abacavir. However, the dysfunction is correlated with high glucose levels (hemoglobin A1c) as well as with hypercholesterolemia in HIV patients but not in HIV negatives. We conclude that tight monitoring and interventions to obtain euglycemia and normocholesterolemia are particularly important in HIV patients to avoid development of autonomic dysfunction. Further studies are needed to elucidate why HIV positives are more sensitive to changes in glucose and lipids.
